# The impact of bio-logging on body weight change of the Eurasian beaver

**DOI:** 10.1371/journal.pone.0261453

**Published:** 2021-12-23

**Authors:** Christian Andre Robstad, Hanna Kavli Lodberg-Holm, Martin Mayer, Frank Rosell

**Affiliations:** 1 Faculty of Technology, Natural Sciences and Maritime Sciences, Department of Natural Sciences and Environmental Health, University of South-Eastern Norway, Notodden, Norway; 2 Department of Bioscience, Aarhus University, Rønde, Denmark; Universidad de Guadalajara, MEXICO

## Abstract

Bio-logging is a common method to collect ecological data on wild animals, but might also induce stress, reduce body condition, and alter behavior. Eurasian beavers (*Castor fiber*) are a semi-aquatic and nocturnal species that are challenging to observe in the wild. Bio-loggers are hence useful tools to study their behaviour and movements, but this raises concerns of potential negative impacts of tagging. To investigate the potential negative impacts of glue-on tags, we compared body weight change for tagged and untagged Eurasian beavers. We hypothesized that tagged beavers would gain less body weight compared to untagged beavers, and that weight change might be affected by tagging length, tag weight, water temperature and the season of tagging. Daily percentage body weight change in relation to initial body weight during the first capture was compared during 57 tagging periods (18±7 days) and 32 controls periods (64±47 days). Body weight change varied between the two groups, with untagged beavers on average gaining daily weight whilst tagged beavers on average lost weight daily, indicating a negative effect of tagging. The average reduction in percentage body weight change per day for tagged beavers was small (0.1 ± 0.3%), and with large individual variation. Neither tag weight, number of tagging days, nor season were important in explaining body weight change of tagged animals. In other words, we found that tagging reduced daily body weight during the tagging period but were unable to determine the mechanism(s) responsible for this decline. Detrimental effects of tagging have important implications for animal welfare and can introduce bias in data that are collected. This calls for careful consideration in the use of tags. We conclude that studies investigating the effects of tagging should consider individual variation in the effects of tagging and, where possible, compare tagged animals with a control group.

## Introduction

Bio-logging studies, i.e. using animal-borne devices to gather information on animal behavior, movement, physiology, and environmental conditions, are key to increasing our understanding of animal behavior and ecology [1, 2]. However, negative impacts of attaching instruments to animals can be substantial, yet are not always considered [3]. Generally speaking, the impact of tag weight is a primary concern, and contemporary guidelines endorse continued miniaturization of tags [4]. The total weight of a tag should not exceed 0.7–10% of body weight depending on the species and recommendations from different authors [5–9]. Moreover, negative effects from bio-logging can be caused by capture stress [8, 10] and vary depending on the attachment method [11]. For example, increased drag from external tags affects energy expenditure, locomotion, and swimming speed [12], especially for aquatic and semi-aquatic species [13–16]. Tagging may negatively impact both body condition and life history traits such as reproduction, parental care, and survival [17, 18]. Such detrimental impacts raise concerns around animal welfare and ethics. In addition, if the body condition, energy expenditure or activity patterns of animals are affected, this can produce biased data that does not reflect the true behaviour of the species [19, 20]. The effects of tag attachment vary among species, individuals [16], and geographic regions [21], and require species-specific adaptations [22]. Impacts from tagging and capture may also vary according to the sex and age of an animal [14, 23]. Assessments should therefore be made to the extent of the tag impact through species-specific studies using different tag types [18]. Importantly, the effects of tagging on animal behaviour and body condition are often not fully understood due to the difficulty in comparing tagged animals with a control group of untagged individuals [24]. This should be investigated and assessed whenever possible [25]. External attachment mechanisms for bio-logging devices such as harnesses, backpacks, collars, and glue-on tags have been used for scientific studies of numerous taxa from invertebrates to mammals [26–29]. The glue-on method is commonly used on animals that are unsuitable to tag with a collar, and involves gluing the tag directly onto the fur or skin of the animal with fast-curing adhesives [30, 31]. However, several studies have found that such tags may impact animals negatively. For example, glue-on tags affected swimming speed due to increased drag in Hawaiian monk seals (*Monachus schauinslandi*) [32], increased trip duration in Antarctic fur seals (*Arctocephalus gazella*) [33], changed foraging behavior in harbour seals (*Phoca vitulina*) [34], and caused skin abrasions on Weddell seals (*Leptonychotes weddellii*) and southern elephant seals (*Mirounga leonina*) [35]. Gluing tags onto the fur is a common method for tagging semi-aquatic mammals. For animals that are insulated by blubber, such as pinnipeds, glue-on tags are expected to have limited impact on body temperature, despite potential heat leakage at the site of tag attachment (e.g. in grey seals) [36]. However, semi-aquatic species that rely on a water-resistant fur for insulation [37] may be more vulnerable to heat loss from glue-on tags that impair the insulating properties of their pelage [38, 39].

The Eurasian beaver (*Castor fiber*) together with the closely related North American beaver (*C*. *canadensis*) are semi-aquatic and nocturnal, which makes it challenging to observe their behavior and habitat use [40, 41]. Therefore, bio-logging is a powerful tool to study their secret lives [42–44]. As ecosystem engineers that have large impacts on their surrounding ecosystems [45, 46], they are often the focus of ecological studies in which bio-loggers are a valuable tool. However, beavers have fusiform body shapes and thick necks with small heads that render the common tagging method of collaring impossible [41, 47, 48]. Other methods to collect data on beavers include surgically implanted transmitters [47, 49, 50], and securing devices using a hole made in the beaver’s tail [51–55]. However, implants can cause extended recovery and surgical complications [56], while tail mounting may cause tail injuries and reduce body weight gain during winter [55]. Due to these challenges, glue-on tags have been utilized as an alternative method for beaver bio-logging by the Norwegian Beaver Project (NBP) [42, 44, 57].

A study investigating short-term changes in post-tagging behaviour of beavers following tagging with glue-on tags, found only minor alterations in behaviour through reduced activity levels of individuals, and four beavers remained in their lodge until the next day following attachment of glue-on tags [58]. Another long-term study within the same population found that the number of captures and handling events negatively affected beaver reproduction during the first years of monitoring, but this effect subsided during the later years of the study, possibly due to habituation of older beavers captured several times throughout their life [59]. The same study found no long-term effects related to whether a beaver had carried a tag in its lifetime on body condition, survival or reproduction [59].

Changes to body condition or body weight in beavers as a result of glue-on tags have not yet been studied during the tagging period. Beavers usually gain body weight from spring to fall and decrease body weight in winter when food availability is scarce [60, 61]. Young beavers tend to gain more body weight than adults, with the weight of beavers peaking at around 10 years of age, and adult females tend to have larger body masses than males in spring and summer [59]. The aim of this study was to investigate potential negative impacts of tagging on the body weight change of beavers by comparing tagged and untagged (control) individuals. We also aimed to identify variables related to tagging that could influence weight change, while controlling for potential effects of year, age, and sex. We hypothesized that glue-on tags would reduce the rate of body weight gain in beavers and predicted that tagged beavers would gain less body weight during the tagging period compared to untagged beavers. Secondly, we hypothesized that negative impacts of tagging on beaver body weight would be exaggerated during the colder months in spring and fall, and at colder water temperatures when beavers spend more energy on thermoregulation, and have lower food access compared to the warmer summer months [45]. Lastly, we hypothesized that the negative effect of tagging on body weight change might be exaggerated by the increased tag weight and duration of the tagging period.

## Materials and methods

### Study area and beaver population

We studied beavers in the NBP study site. This site consists of three connected rivers–Gvarv (59° 386’ N, 09° 179’ E), Straumen (59° 29’ N, 09° 153’ E), and Sauar (59° 444’ N, 09° 307’ E)–which all discharge into lake Norsjø in Vestfold and Telemark County, Norway. The mean annual air temperature is 4.6 Celsius and annual precipitation is 790 mm [59, 62]. Human settlements, semi-agricultural landscapes, and forested woodlands are scattered throughout the study area [62]. Eurasian beavers have inhabited the area since the 1920s [63]. Hunting pressure in the area is low [64], and the population is considered to be at carrying capacity [42, 62, 64]. Beaver territories border each other, so there are few unoccupied stretches of the river. In total, 25–30 beaver families inhabit the study area and they have been continuously monitored since 1997 [42, 65, 66].

### Capture and handling, and details of study individuals

We studied 57 beavers that had been captured as part of ongoing research. Specifically, every year between March and November, beavers were captured as part of a long-term capture-mark-recapture study and for tagging purposes [65, 67]. The NBP has studied beavers for over 20 years with 1,560 live captures. We attempted to reduce capture-related stress by not capturing the same beaver several times a year, which resulted in a lower sample size of untagged beavers (control group) compared to tagged beavers. Beavers were caught at night with nets from a boat or onshore [68], and transferred into a cloth bag for handling. All beavers included in this study were handled while awake with no use of anesthesia. Captured animals were microchipped and ear-tagged for identification [69]. Each beaver was sexed, based on the color and viscosity of the anal-gland secretion [70], weighed (tagged beavers were weighed with the tag during attachment, and without the tag after removal) and measured for body size, tail length, and tail thickness [65, 71]. Beavers were subsequently released back to the place of capture after 20–40 minutes. Age was assigned based on body weight if not captured for the first time as a kit or yearling. Individuals captured for the first time weighing between >17 and <19.5 kg were assigned a minimum age of two years and a minimum age of three years when over >19.5 kg [72]. We classified the beavers into age groups based on prior knowledge of rate of body weight change for different ages. Beavers aged between 2–3 years were defined as ‘young’ (i.e. individuals that are still growing), those between 4–10 were classified as ‘adult’ (fully grown with a lower body weight gain compared to young beavers). Beavers aged 11–17 were classified as ‘old’ (showing signs of senescence, such as loss of body weight). The average age when beavers in our study population tend to die or disappear is 9–10 years independent of their sex [67], but beavers in the wild can live up to 20–24 years of age [73–75]. We have had several beavers older than 10 years in our study area, with a maximum age of 17 included in the dataset [65, 76].

### Tagging procedure

Since 2009, glue-on tags have been the main attachment method under the NBP. From 2009 to 2020, beavers were equipped with tags sized approximately 12 × 12 x 1.2 cm with tag weight varying according to logger weight and the materials used (total logger weight: 159 ± 36 g, range: 124–267 g). Every tag unit consisted of a VHF transmitter (18 × 35 mm, 10 g; Reptile glue-on series R1910; Advanced Telemetry Systems, Isanti, MN, USA) in combination with a global positioning system (GPS) device–model G1G 134A; Sirtrack, Havelock North, NZ (50 × 70 mm, 24 g), or model Gipsy 5 GPS; TechnoSmart, Rome, Italy, (48×21 mm, 27.8 g). Most beavers were also equipped with triaxial accelerometers (15 × 90 mm, 62 g; JUV Elektronik, Schleswig-Hollstein, GER) or a Daily Diary (52 x 29 x 22 mm, 62 g with waterproof casing) developed by the SLAM lab at Swansea University, UK. During a few tagging sessions, a time-depth recorder (67 x 17 mm, 30 g; model MK9 Archival Tag, Wildlife Computers Inc, Redmond, WA, USA) was used instead of a GPS or accelerometer. The loggers were attached to a piece of coarse polyester, secured with cable ties and glue (both to the polyester and to each other), to assure all parts remained in one unit until retrieval of the tag.

**Fig 1:**
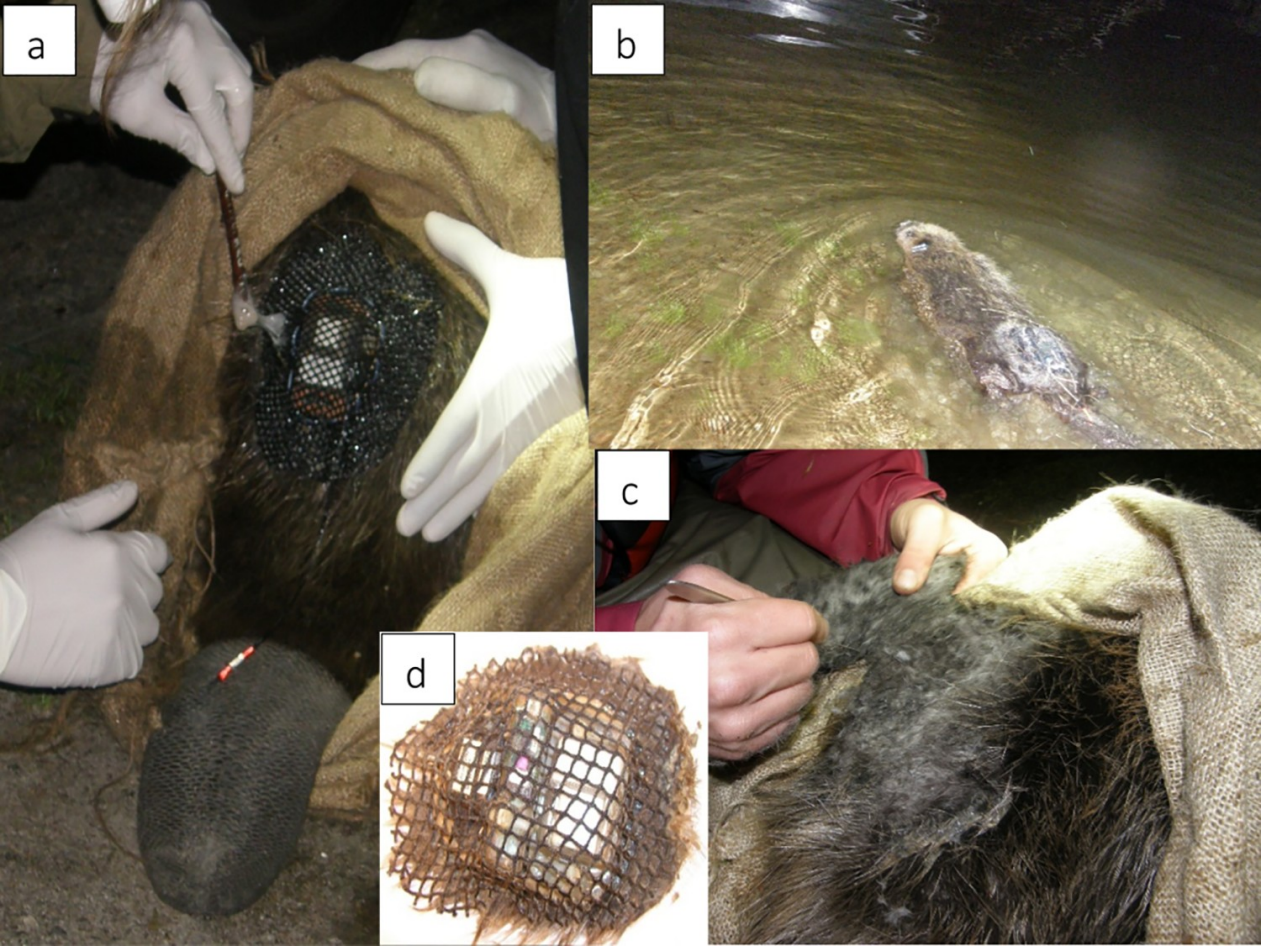
Process of tagging a Eurasian beaver (*Castor fiber*) using glue-on tags within the study area in southeastern Norway. Attachment of the tag using two-component epoxy glue (a), release of the beaver with the tag attached (b), removal of tag by cutting it out of the outer fur using a scalpel (c), and the tag following removal (d). Photos: Patricia Graf.

The tag was glued onto the lower back of the beaver, 15 cm above the base of the tail irrespective of beaver body size, using a two-component epoxy resin (System Three Resins, Auburn WA, USA) [58] (Fig 1). Note that tags were covered with a 4.5 mm mesh net (Mørenot Fishery AS, Møre and Romsdal) on both sides to prevent the glue from reaching the skin of the beaver which may cause heat or chemical burns (Fig 1). After attaching the tag to the beaver, we waited for the glue to harden, and rinsed the beaver and tag with cold water if we registered a rise in temperature that may harm the beaver. Temperatures above 50°C cause damage to the skin of animals [77], but laboratory analysis using similar glues as we applied, showed that three types of epoxy glue never reached temperatures above 34°C [35]. We monitored the temperature of the glue with our hands while wearing latex gloves. The whole capture, handling and attachment process took between 20–40 minutes. Tagged beavers were usually recaptured after 2–3 weeks, and the tag was cut out of the fur using a scalpel, which took approximately 10–40 minutes. We defined the tagging period from the time the tag was attached on the beaver until the beaver was recaptured and the tag removed. If the tag fell off by itself, the beaver was not recaptured, and the body weight difference from the beginning to the end of the tagging period could not be estimated. These tagging events were therefore not included in the analysis. The weight of each tag was estimated by adding together the weights of the individual loggers and an average weight of the materials used for attachment. We weighed 21 tags post-removal (and after having removed the data loggers) to estimate the average weight of the glue and materials used for attachment; this was 90 ± 27 g, and we therefore added this as a constant to each tag weight.

### Ethical statement

Ethical committees within the Norwegian Food Safety Authority (most recent authorization FOTS ID 15947) and the Norwegian Directorate for Nature Management (most recent authorization 2014/14415 ART-VI-ID) approved this study, including all handling and tagging procedures. To our knowledge, none of the beavers in this study were injured due to capture and handling. Unfortunately, one beaver was found deceased in the entrance of the lodge during the tagging period but cause of death and whether it was related to tagging could not be determined based on a necropsy by the Norwegian Veterinary Institute. The study complied with the ASAB/ABS Guidelines for the treatment of animals in behavioral research and teaching [78]. The study was also carried out in compliance with the ARRIVE guidelines [79].

### Data preparation

We used percentage body weight change between initial and the subsequent capture of tagged and untagged beavers as an indicator of change in body condition. Tagged beavers were included if their body weight was measured both when the tag was attached (capture) and removed (recapture). Untagged beavers were only included in the control group if captured twice between March–November in a given year, in order to calculate their daily body weight change between the two captures. We first estimated total body weight change (%) between the two captures, and then divided this by the number of days between capture and recapture to obtain daily body weight change (%) in relation to the initial body weight (at first capture). The percentage weight of the tag in relation to the initial beaver body weight was also calculated, which we hereby refer to as relative tag weight (%).

Glue-on tags were only used on beavers after 2009, so we only included observations of tagged beavers during or following that year (2009–2020), while observations of untagged beavers were included from 2006–2020. Prior to 2006, the beavers were weighed with less accuracy, and these observations were therefore not included. Four of the female territory owners included in the study were pregnant in the period between captures. We conducted our analysis both with and without these individuals included, and because they did not impact the model selection or model estimates, they were retained in the dataset. Similarly, four tagged beavers were older than the maximum age of the untagged beavers (13 years old), which increased the average age of the tagged beavers. We also tried to exclude these older tagged beavers from the analysis, but this did not influence model selection or model estimates, and they were therefore retained in the dataset. Most of the study beavers have been monitored over many years, and some were included several times in the dataset, either as tagged or untagged (S1 File).

We defined season according to the months in which the majority of the days between captures fell within. Observations with the majority of days during March-May were defined as spring, June-August as summer, and September-November as fall. We obtained average water temperature measurements in Celsius from the Norwegian Water Resources and Energy Directorate (NVE) for each river system during the periods beavers carried the tags. Water temperatures were not monitored within the study area itself, and we therefore had to rely on data from the same watershed to indicate water temperature. For Sauar, we used water temperature data from Kirkvoll located on the Tinnåa river that runs into Heddal lake, which represents the start of Sauar river. For Straumen, we obtained water temperatures from Kilen in the Kilåi river than runs into Flåvatn, which represents the start of the Straumen river. For Gvarv, we used water temperature information from the Hørte river, which merges with the Gvarv river just above the study area.

### Statistical analysis

We divided the analysis into two parts. First, we modelled the daily body weight change (%) as a function of tagging status (tagged or untagged), including age group, season and sex as possible confounding variables. Second, for tagged beavers, we modelled total body weight change (%) in relation to initial weight as a function of tagging season, relative tag weight (%), average water temperature during the tagging period, sex, age group, and the duration of tagging. We excluded the six tagged beavers for which we could not obtain water temperature data in this analysis. For both analyses, we used linear mixed models (LMM) with a Gaussian distribution and the R package lme4 [80]. Beaver ID and year were included as crossed random effects on the intercept in both analyses to account for pseudoreplication of repeated sampling of the same individual and yearly variations. In part one of the analysis we included interactions between the tagging status of the beaver (tagged or untagged) and its sex, age group and the season of tagging. The recommended steps from Zuur, Ieno and Elphick [81] were followed for data exploration. All numerical variables were tested for collinearity and were found not to be correlated (Pearson r coefficient <0.6) and with a cut-off of variance inflation factor (VIF) < 3 [82]. We visually assessed collinearity between numerical and categorical variables using boxplots. The beavers’ social status was collinear with age and was therefore excluded from the analysis. Season of tagging and water temperature were also collinear, so we included them both in different models.

We constructed a set of candidate models with separate combinations of the explanatory variables for both parts of the analysis. Model selection was based on Akaike’s Information Criterion (AICc) values for small sample sizes [83, 84], and carried out with the R package MuMln using the "model.sel" function [85]. To avoid selecting complex models that add little additional information compared to a similar nested model, we selected the most parsimonious model among the candidate models separated with less than <2 ΔAICc from the top model [86, 87]. For each explanatory variable included in the most parsimonious model, we calculated the 95% confidence interval, and if it overlapped with zero, the variable was considered uninformative. We plotted the Pearson residuals of the most parsimonious model against fitted values to inspect for non-normality and heterogeneity [88]. We also plotted the model residuals against each variable included and not included in the most parsimonious model, and fitted a smoother to check for potential non-linear patterns in the residuals. Lastly, we simulated residuals from the model using the package DHARMa and plotted the residuals against expected and fitted values to observe deviations from the expected distribution [89]. All data analyses were performed with R version 4.0.3 (R Development Core Team 2020).

## Results

**Fig 2:**
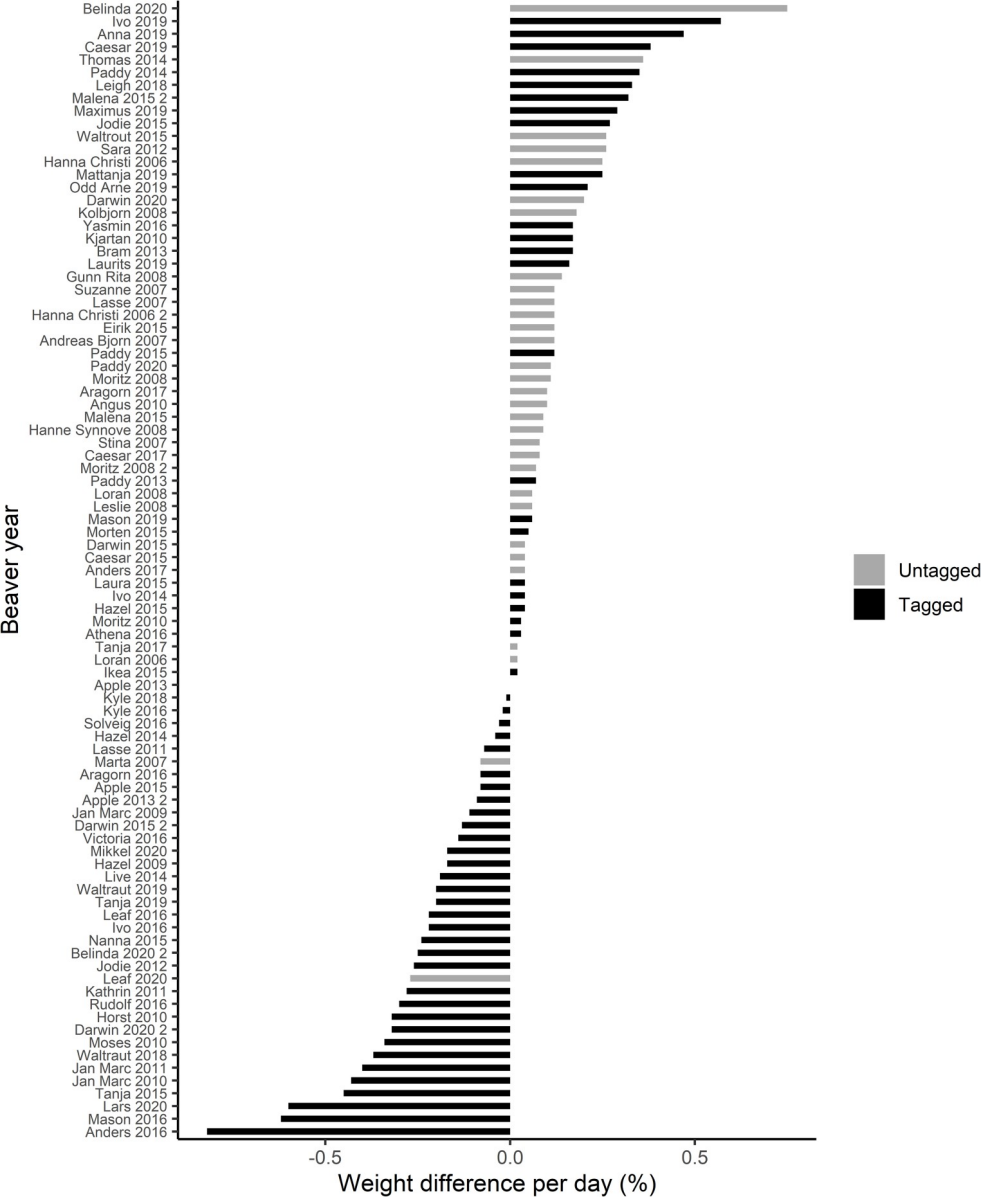
Daily percentage body weight change between captures for Eurasian beavers (*Castor fiber*) in southeastern Norway 2006–2020. Tagged beavers are shown in black while untagged are shown in dark grey.

We analyzed beaver body weight change across 89 periods (tagging periods or between captures of untagged beavers) of 57 individual beavers (S1 File). We included 57 tagging periods (20 females, 22 males), and 32 control periods (14 females, 13 males). Of these,11 beavers were tagged more than once, while 13 were included with more than one control period, and 13 beavers were included as both tagged and controls at different times (S1 File). The mean age of the two groups differed by 1.8 years, and the mean number of days between captures was 18 ± 7 days for tagged beavers and 64 ± 47 days for untagged (Table 1). Water temperature varied among tagging periods between 1.29 to 15.87 Celsius. The relative tag weight (%) constituted 0.8 ± 0.2% (range, 0.5–1.4%) of the initial body weight of the beavers. Between captures, untagged beavers gained an average of 23 g per day, while body weight of the tagged beavers declined by 13.7 g daily (Table 1). Percentage daily body weight change was on average 0.1% for untagged and -0.1% for tagged beavers, but with considerable individual variation (Fig 2). The largest decline in body weight among the tagged beavers was for an individual that lost 2,333 g (11.5% reduction) over a 14-day tagging period; this constituted 0.8% daily weight loss. Amongst the tagged beavers, 57% of the tagging periods caused a decline in body weight, but 42% of the tagged beavers gained body weight. Two (7%) of the untagged beavers declined in body weight between captures, while 93% gained body weight. Visual observations during tag removal indicated that the amount of fur remaining post-tagging varied. The extent was not documented consistently, but we believe it had a negligible impact on measured body weight. For some beavers, underfur appeared intact, while others had open patches with no underfur remaining.

**Table 1:** Overview of the two Eurasian beaver (*Castor fiber)* groups (tagged and untagged) in relation to number of days between captures, and total and daily body weight change between the captures in southeastern Norway 2006–2020. Each variable is presented as the mean with standard deviation and the total range within the parenthesis.

	Tagged	Untagged
**Days between captures**	18 ± 6.6 days (8–43)	64 ± 46.8 (5–139)
**Age**	6.7 ± 3.5 years (2–17)	4.9 ± 3.0 years (2–13)
**Average total body weight change**	-220.7 ± 953.5 g (-2,333–2,077.8)	1,156.9 ± 953.5 g (-500–3,500)
**Average daily body weight change**	-13.7 ± 59.4 g (-166.64–115.43)	23 ± 3.0 g (-66.67–149.63)
**Average daily body weight change %**	-0.1 ± 0.3% (-0.82–0.57)	0.1 ± 0.2% (-0.27–0.75)

### Daily weight change (%) in tagged versus untagged beavers

**Fig 3:**
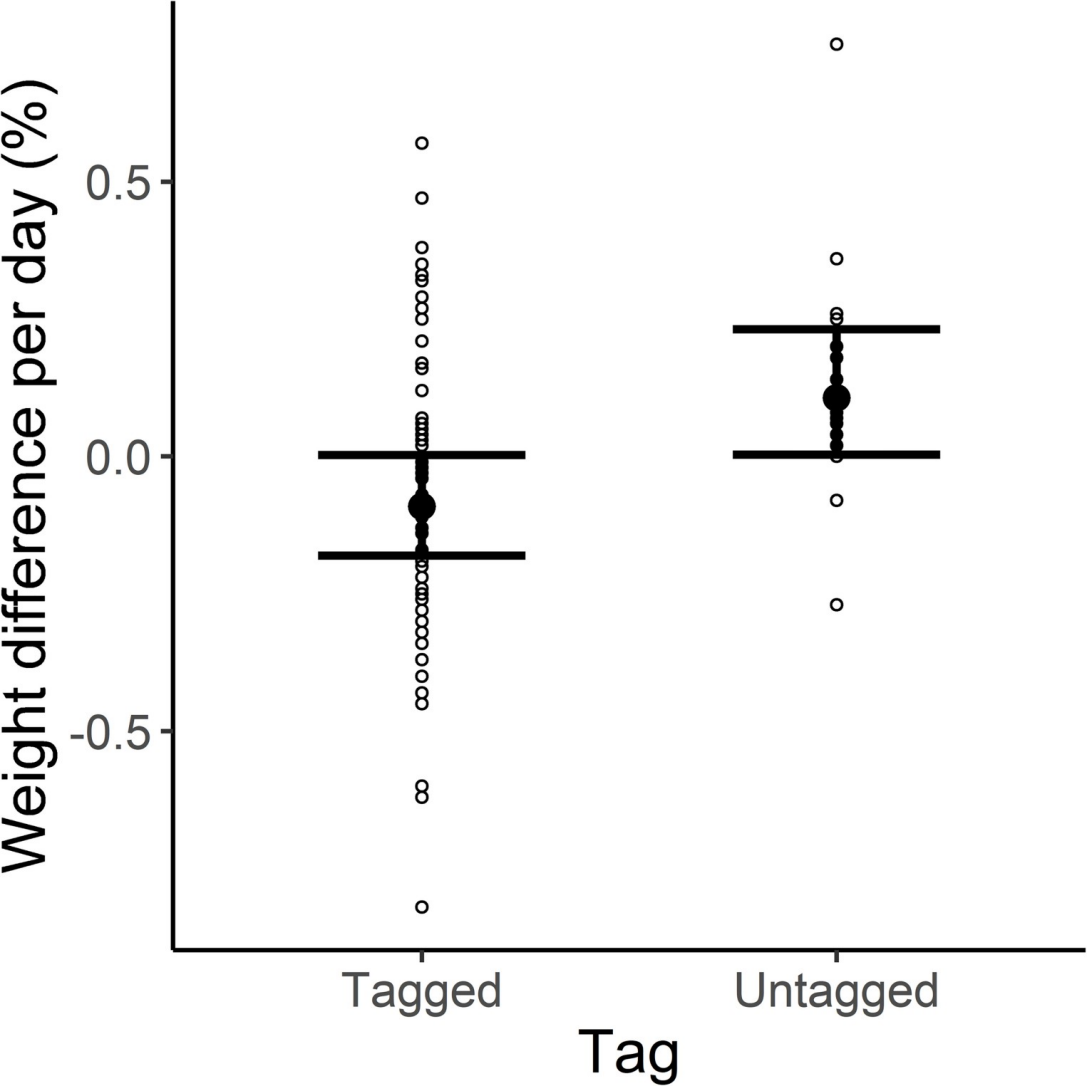
Estimated daily weight change (%) between captures for tagged and untagged Eurasian beavers (*Castor fiber*) in southeastern Norway (2006–2020). The prediction is based on a linear mixed effects model using percent daily body weight change as the response variable. The horizontal lines represent the 95% confidence intervals around each mean.

The most supported model retained tag presence and age group as fixed effects (Table 2). Predicted daily body weight change of tagged beavers was -0.04 g (CI: -0.18–0.09), while predicted daily body weight change of untagged beavers was 0.20 g (CI: 0.07–0.32; Fig 3 & Table 3). Age group was uninformative (95% confidence intervals overlapped zero) in explaining daily body weight change for tagged and untagged beavers (Table 3). The interactions between tag presence and either sex or season were unsupported. The diagnostic plots indicated acceptable model fit using a Gaussian distribution (S1 File).

**Table 2:** The model selection results investigating the percentage daily body weight change between two subsequent captures of Eurasian beavers (*Castor fiber*) in southeastern Norway (2006–2020). Each model represents a linear mixed effect model with year and beaver ID included as random effects. Potential fixed effects included in each candidate model are age group of the beaver, season of tagging, sex of the beaver, and whether the beaver was tagged or not. The plus signs indicate which variables were included in each candidate model. Degrees of freedom (df), log likelihood (logLik) and model AIC weight (w) are provided for each candidate model. Results for the best supported model are shown in bold.

Intercept	AgeG	Season	Sex	Tag	AgeG*Tag	Season *Tag	Sex *Tag	df	logLik	AICc	ΔAICc	w
**-0.04**	**+**			**+**				**7**	**4.36**	**6.66**	**0.00**	**0.90**
0.00								4	-2.27	13.01	6.35	0.04
0.08	+							6	-0.47	13.96	7.30	0.02
-0.09	+	+	+	+		+		12	6.55	15.01	8.34	0.01
0.09	+		+					7	-0.39	16.16	9.49	0.01
0.03	+	+						8	0.48	16.83	10.17	0.01
-0.08	+	+	+	+		+	+	13	6.57	17.72	11.06	0.00
-0.08	+	+	+	+		+	+	13	6.57	17.72	11.06	0.00
-0.11	+	+	+	+	+	+		14	6.76	20.15	13.49	0.00
-0.11	+	+	+	+	+	+	+	15	6.76	23.05	16.39	0.00

**Table 3:** Effect size (β), adjusted standard error (SE), lower (LCI), and upper (UCI) 95% confidence interval of explanatory variables in the most parsimonious model analyzing percentage daily body weight change between two subsequent captures of Eurasian beavers (*Castor fiber*) in southeastern Norway (2006–2020). The intercept includes the ‘young’ age group of beavers. The informative parameters are shown in bold, and the marginal (R^2^m) and conditional (R^2^c) R squared is given for the model overall.

	β	SE	LCI	UCI	R^2^m	R^2^c
Intercept (Tagged)	‒0.04	0.07	‒0.18	0.09	0.16	0.40
**TagUntagged**	**0.20**	**0.06**	**0.07**	**0.32**		
AgeGroup (Adult)	‒0.05	0.06	‒0.16	0.07		
AgeGroup (Old)	0.00	0.09	‒0.18	0.18		

### Total weight change (%) in tagged beavers between tag attachment and removal

The null model was the most supported model; none of the explanatory variables (relative weight of tag (%), average water temperature during the tagging period, sex, age group, season, and length of the tagging period) were retained in the model selection to explain total body weight change (%) (Table 4).

**Table 4:** The model selection result, investigating percentage body weight change during tagging of Eurasian beavers (*Castor fiber*) in South-Eastern Norway (2009–2020). Each model represents a linear mixed effects model with beaver ID included as a random effect. Potential fixed effects included in each candidate model are tag weight as a percent of initial beaver body mass (tag weight %), age group of the beaver, number of days tagged, season of tagging, sex, and average water temperature during the tagging period. The plus signs indicate the categorical variables included, and values are given for numerical variables that were included in each candidate model. The intercept includes the ‘young’ age group of beavers. Candidate models were ranked based on AICc, and the range of ΔAICc <2 are shown in bold. Degrees of freedom (df), log likelihood (logLik) and AIC weight (w) is provided for each candidate model.

Intercept	Age group	Relative tag weight %	Tagging days	Season	Sex	Average water temperature	df	logLik	AICc	delta	w
**‒1.44**							**4.00**	**‒144.08**	**297.03**	**0.00**	**0.78**
‒1.59	+						6.00	‒143.96	301.83	4.80	0.07
‒3.22	+		0.09				7.00	‒143.37	303.35	6.32	0.03
‒0.37	+					‒0.14	7.00	‒143.47	303.53	6.50	0.03
‒0.14	+	**‒**2.12					7.00	‒143.75	304.10	7.07	0.02
‒1.40	+				+		7.00	‒143.89	304.39	7.36	0.02
‒2.05	+		0.10			‒0.14	8.00	‒142.79	305.01	7.98	0.01
‒1.54	+			+			8.00	‒143.22	305.88	8.85	0.01
‒1.90	+	‒1.75	0.09				8.00	‒143.23	305.88	8.85	0.01
‒3.36	+		0.10	+			9.00	‒142.66	307.70	10.67	0.00
‒1.93	+		0.10		+	‒0.14	9.00	‒142.73	307.86	10.83	0.00
‒1.79	+	‒1.65	0.09		+		9.00	‒143.15	308.70	11.66	0.00
‒1.81	+	‒2.10	0.09	+			10.00	‒142.44	310.39	13.36	0.00
0.07	+	‒2.46	0.09		+	‒0.16	10.00	‒142.46	310.42	13.39	0.00
‒1.79	+	‒2.00	0.09	+	+		11.00	‒142.41	313.59	16.56	0.00

## Discussion

We investigated the effects of glue-on tags on body weight change of Eurasian beavers. We found support for our first hypothesis that tagging reduced beaver body weight gain during the tagging period (and actually led to body weight loss) compared to untagged beavers that generally gained body weight. Our hypothesis that tagged beavers should gain less body weight during the colder months of the year or with colder water temperature was unsupported. Moreover, we found no evidence that relative tag weight or the number of tagging days affected beaver body weight. The tagged beavers did, however, display individual variation with some tagged beavers gaining body weight, while most lost body weight during tagging. The average daily weight loss in tagged beavers of 13 g (0.1%) appears small, but importantly indicates that tagged beavers were (on average) unable to gain weight compared to untagged beavers that gained 23 g (0.1%) daily. This difference between the two groups, as well as some tagged beavers displaying considerable declines in body weight, indicates that tagging can have a negative impact on beavers.

### Control groups

We included a control group of untagged beavers in our study, who mostly increased daily body weight between captures in line with previous studies [61]. Comparing tagged animals with a control group may help quantify the impact of tagging and avoid conclusions based on statistical artifacts that may arise without one [90]. While the weight loss of tagged beavers in our study was small on average, this was in direct contrast to untagged beavers who saw a small increase in body weight. Many bio-logging studies lack a control group due to the challenges with monitoring wild animals that are not tagged, which may influence the conclusions of these studies. Other semi-aquatic mammals such as the Eurasian otter (*Lutra lutra*) [91] and platypus (*Ornithorhynchus anatinus*) [92] tagged with glue-on tags displayed no apparent adverse effects. However, the authors did not compare the tagged animals with an untagged control group, which may impact the results.

Defining a true control group to include in wildlife studies can bring additional challenges. In the current study, there was a large difference in the number of days between the captures in the control group (mean = 64 days) and the tagged individuals (mean = 18 days). This could potentially lead to biases, requiring a careful interpretation of the results. In wild populations, it can be challenging to capture a sufficient number of individuals (both for logistic and economic reasons) to have a control group that is directly comparable to the tagged animals. Here, we accounted for confounding variables such as age group, sex, season, year and individual variation. Despite controlling for these, we failed to identify any variables related to the tagging process aside from the presence of a tag that drove the observed negative effects, suggesting further investigation is needed.

### Relative tag weight and drag

The decline in body weight of tagged beavers was not exaggerated by the relative weight of the tag. If added weight was the main cause for the lower body weight gain, we would expect beavers that carried heavier tags, or carried the tag over a longer period to display the strongest decline in body weight. The tags made up between 0.5–1.3% of the initial body weight of the tagged beaver, which is well below both the 3% and 5% limits suggested by several studies [7]. A meta-analysis reviewing 214 studies of birds tagged using various tag types and attachment methods, found that negative effects of tagging were only apparent when tags weighed more than 1% of the bird’s body weight [17]. As most of our tags were below this 1% threshold, we would not expect to observe a strong effect due to relative tag weight.

The shape of the tag and its location on the body of an animal may be more important than tag weight, as these factors directly impact tag-induced drag; something that is essential for animals moving in fluid media [12, 93]. For example, drag created by an external tag might increase energy expenditure, thereby increasing foraging time [94, 95]. A simulation study on grey seals (*Halichoerus grypus*) showed that shape of a tag is much more important than its size, e.g. a larger but more streamlined tag can reduce drag by 22% in swimming seals compared to a smaller but conventionally shaped tag [93]. We did not record the size or shape of the tag attached to beavers, and can therefore not directly analyze the drag impact. However, the relatively slow swimming speed of Eurasian beavers compared to other aquatic or semi-aquatic species may make them less susceptible to negative impacts from tag-induced drag. Indeed, drag is influenced by the speed and acceleration of the tagged animal, with slower moving animals not relying on high acceleration to capture prey being less impacted [9]. Great cormorants (*Phalacrocorax carbo*) were only negatively affected by drag from a tag at swimming speeds >1.4 m/s [4]. Beavers rarely swim faster than 0.8 m/s when swimming at the surface [96], and 0.6 m/s when diving [97]. The tags used on beavers here are usually above the surface of the water when beavers are swimming, and the beavers spend only 3% of their active time diving [57]. Thus, we speculate that the tag-induced drag impact of the glue-on tags used here may have been relatively small. However, we cannot make certain conclusions regarding drag in tagged beavers, and future studies should attempt to address this.

### Tagging season and tag removal

The season of tagging did not impact body weight change of the beavers, and neither did water temperature in the river. A study of the impacts of tags on North American beavers in Minnesota, USA, that used different tagging methods (tail tags and implants) found no difference in body weight between tagged and untagged individuals during summer, but tagged animals lost more body weight in winter [55]. We did not tag beavers during the winter months, which might partly explain why we did not observe a seasonal effect. Beavers may be more susceptible to negative tagging effects during winter due to the colder winter temperatures and lower access to green plants during this time. In ice free areas beavers may even increase their active time and time spent foraging during winter, and tagging during this time may represent an additional stressor [96]. Semi-aquatic species are in an especially energetic precarious position due to changing temperature conditions in water and on land [37]. A major concern with attaching a tag to the fur of a semi-aquatic animal is how it impacts the thermal insulation properties of the fur [36]. Beaver fur consists of a protective overlayer of coarse guard hairs and an underlayer of fur-wool [98], functioning as a thermal barrier in both air and water [99]. Beavers groom regularly to maintain the waterproof properties of the fur [100, 101] and a glued patch will impair their ability to groom that patch, which might cause heat loss. Additionally epoxy used to attach the tag has a higher thermal conductivity than fur, and therefore may act as a source of increased energy expenditure [36]. Grey seals tagged with glue-on tags displayed no apparent heat loss when wet, but heat loss was clear when animals were dry [36]. We would recommend that future studies apply thermal imaging to measure differences in surface temperature of tagged beavers both when they are wet and dry.

Following tag removal, we observed cases in which the underfur was left intact, but also occasions where patches with no underfur were left on the beaver. Fur loss may be exaggerated by excessive amounts of glue and attachment too close to the skin. The damaged patch left after tagging beavers with glue on tags usually takes 3 to 4 months to be restored [58]. Thus, the open patch may be a source of heat loss until the fur is regrown, further reducing body weight because resources are invested into thermoregulation. Future studies should explore whether the negative effects on body weight continues after tag removal. Skin irritation or major wounds following tagging has not previously been documented with glue-on tags on beavers, with the exception of one beaver included in our study with a small wound underneath the logger. Tagging of nutria (*Myocastor coypus*) using glue-on tags on the tail caused sloughing of the skin [102], which we did not observe in our beavers. There is always a trade-off between minimizing the amount of glue in order to reduce damage to the beaver fur, and so minimize the compromise to thermal insulation and using sufficient amounts to ensure that the tag remains attached to the beaver for the required duration.

### Tagging length, handling and individual variation

The length of the tagging periods varied between 8 to 43 days. While we attempted to recapture beavers after 2–3 weeks for tag removal, some were retrieved earlier due to different battery capacities of the loggers. Some beavers also evaded capture and carried the tag for longer time periods. Despite the considerable variation, we did not find that tagging length affected body weight change. A comprehensive long-term study in the same population of Eurasian beavers found that beaver body mass and litter size (in old individuals only) decreased with increasing number of captures [59]. However, this effect was less clear during the later years of the project, indicating possible habituation towards capture and handling [59]. A previous tagging study from the same area also found that tagged beavers tend to spend more time in the lodge during the night they were tagged compared to later in the tagging period [58]. Upon capture, tagged beavers are typically handled for longer time periods than non-tagged beavers, due to the extra time required to attach and remove the tag. However, as we cannot monitor the spatial movements of untagged beavers, we cannot conclude on whether this behavior is driven by the capture itself or related to carrying the tag. Future tagging studies should attempt to reduce handling time during tagging as much as possible to mitigate any potential negative impacts.

We found considerable individual variation among the tagged beavers, and while the majority lost body weight during tagging, some tagged beavers also gained body weight. In comparison only two of the untagged beavers lost body weight between captures. This indicates that it is unusual for beavers to lose body weight between spring and autumn (March and November). Thus, body weight loss in 57% of the tagged beavers is a cause for concern. Habituation to both capture and tagging could go some way to explain why some tagged beavers gained body weight, and this would be interesting to explore in future studies with additional data on body weight gain of the same individuals during several tagging periods.

### Conclusion

Our study emphasizes the importance of using a control group to investigate tagging effects on wildlife. We found a negative effect on beaver body weight change during the tagging period, but with considerable individual variation amongst tagged individuals. These findings might have implications regarding animal welfare and the validity of data collected [103, 104]. The large variation among tagged beavers illustrates how effects of tagging should not only be studied by comparing averages among tagged and untagged individuals, but also by analyzing individual impacts. The negative effect of tagging did not vary as a function of the confounding variables (sex, age, relative tag weight, tagging duration, season, water temperature) we examined. This means we found no support for our hypothesis that relative tag weight would negatively impact tagged beavers, but the drivers of the observed body weight loss should be investigated further. The observed changes in body weight might be caused by stress associated with capture [105, 106] as tagging usually requires longer handling time, and such impacts on body condition do not necessarily mean that the beavers change their activity patterns or spatial movements [58], or prolonged effects after tag removal, but this should be investigated further. Especially the duration of weight loss in tagged beavers remains uncertain, and this should be explored in relation to environmental conditions and food availability that may exaggerate negative effects during tagging. Glue-on tags are used as a relatively short-term tagging method on beavers, and the cumulative body weight loss observed was not extensive in comparison to their total body weight. Nevertheless, any decrease in body weight gain during tagging may be important in terms of individual animal welfare especially considering that untagged animals by comparison tended to increase their body weight. Assessing potential negative impacts of tagging on both a population and individual level should therefore be a priority for all research projects applying tags on wild animals.

## Supporting information

S1 File This file contains all the supporting tables and figures. (DOCX)
