# Supplementary material for: The impact of bio-logging on body weight change of the Eurasian beaver
Source: PLoS One. 2021 Dec 23;16(12):e0261453. doi: 10.1371/journal.pone.0261453 (PMC8699976; doi:10.1371/journal.pone.0261453)
Supplement: S1 File — (DOCX) [file pone.0261453.s001.docx]

**Supporting information**

Table S1: Overview of tagging periods and control periods of Eurasian beavers (*Castor fiber*) in southeastern Norway (2009-2020). Name of the beaver, age, days between subsequent captures, initial weight at first capture, season and whether it was tagged or not is provided for every period. Also included is how many tagging periods and control periods included for each beaver.

| **Name** | **Age** | **Tagged** | **Number of  tagging periods** | **Number of  control periods** | **Days between  captures** | **Initial weight (kg)** | **Season** |
| --- | --- | --- | --- | --- | --- | --- | --- |
| Anders | 5 | No | 1 | 1 | 108 | 19.80 | Summer |
| Anders | 4 | Yes | 1 | 1 | 14 | 20.23 | Spring |
| Andreas Bjorn | 3 | No | 0 | 1 | 93 | 18.60 | Summer |
| Angus | 2 | No | 0 | 1 | 68 | 16.00 | Summer |
| Anna | 7 | Yes | 1 | 0 | 15 | 23.12 | Spring |
| Apple | 3 | No | 2 | 1 | 28 | 21.50 | Autumn |
| Apple | 3 | Yes | 2 | 1 | 19 | 21.38 | Autumn |
| Apple | 5 | Yes | 2 | 1 | 20 | 20.83 | Spring |
| Aragorn | 3 | No | 1 | 1 | 132 | 18.80 | Summer |
| Aragorn | 2 | Yes | 1 | 1 | 31 | 22.38 | Autumn |
| Asle | 4 | No | 0 | 1 | 116 | 18.00 | Spring |
| Athena | 6 | Yes | 1 | 0 | 12 | 21.93 | Spring |
| Belinda | 3 | No | 1 | 1 | 6 | 20.02 | Autumn |
| Belinda | 3 | Yes | 1 | 1 | 12 | 20.92 | Autumn |
| Bram | 14 | Yes | 1 | 0 | 19 | 19.38 | Summer |
| Caesar | 3 | No | 1 | 2 | 139 | 23.50 | Summer |
| Caesar | 5 | No | 1 | 2 | 108 | 20.70 | Summer |
| Caesar | 7 | Yes | 1 | 2 | 14 | 17.52 | Spring |
| Carl | 5 | No | 0 | 1 | 125 | 17.00 | Summer |
| Chris | 5 | No | 0 | 2 | 83 | 20.00 | Summer |
| Chris | 5 | No | 0 | 2 | 84 | 19.00 | Spring |
| Darwin | 5 | No | 2 | 2 | 126 | 20.00 | Summer |
| Darwin | 10 | No | 2 | 2 | 65 | 18.90 | Summer |
| Darwin | 5 | Yes | 2 | 2 | 15 | 19.38 | Spring |
| Darwin | 10 | Yes | 2 | 2 | 22 | 20.32 | Summer |
| Eirik | 3 | No | 0 | 1 | 13 | 19.50 | Spring |
| Franky | 6 | No | 0 | 1 | 58 | 23.00 | Summer |
| Frode | 5 | No | 0 | 2 | 103 | 21.50 | Spring |
| Frode | 5 | No | 0 | 2 | 31 | 22.50 | Summer |
| Froydis | 4 | No | 0 | 1 | 111 | 19.50 | Spring |
| Gronn | 5 | No | 0 | 1 | 110 | 20.50 | Summer |
| Gunn Rita | 4 | No | 0 | 1 | 16 | 22.00 | Summer |
| Hanna Christi | 3 | No | 0 | 2 | 23 | 20.50 | Autumn |
| Hanna Christi | 3 | No | 0 | 2 | 133 | 17.60 | Summer |
| Hanne | 3 | No | 0 | 1 | 62 | 22.00 | Summer |
| Hanne Synnove | 4 | No | 0 | 1 | 71 | 22.00 | Summer |
| Harald | 5 | No | 0 | 1 | 69 | 21.50 | Summer |
| Havar | 3 | No | 0 | 1 | 46 | 19.00 | Spring |
| Hazel | 5 | Yes | 4 | 0 | 43 | 24.27 | Autumn |
| Hazel | 9 | Yes | 4 | 0 | 9 | 20.83 | Spring |
| Hazel | 10 | Yes | 4 | 0 | 39 | 22.33 | Autumn |
| Hazel | 11 | Yes | 4 | 0 | 21 | 21.83 | Spring |
| Helgenen | 4 | No | 0 | 1 | 86 | 20.00 | Summer |
| Horst | 6 | Yes | 1 | 0 | 18 | 22.83 | Autumn |
| Ikea | 13 | Yes | 1 | 0 | 17 | 22.73 | Autumn |
| Ivo | 4 | Yes | 3 | 0 | 14 | 20.88 | Spring |
| Ivo | 6 | Yes | 3 | 0 | 14 | 18.98 | Spring |
| Ivo | 9 | Yes | 3 | 0 | 18 | 20.42 | Summer |
| Jan Marc | 5 | Yes | 3 | 0 | 20 | 22.37 | Spring |
| Jan Marc | 6 | Yes | 3 | 0 | 20 | 22.83 | Autumn |
| Jan Marc | 6 | Yes | 3 | 0 | 15 | 21.93 | Summer |
| Jobu | 4 | No | 0 | 1 | 103 | 18.00 | Summer |
| Jodie | 14 | Yes | 2 | 0 | 13 | 24.83 | Spring |
| Jodie | 17 | Yes | 2 | 0 | 22 | 19.83 | Spring |
| Jon | 5 | No | 0 | 2 | 154 | 20.50 | Summer |
| Jon | 3 | No | 0 | 2 | 145 | 21.00 | Summer |
| Jorn | 5 | No | 0 | 2 | 132 | 20.00 | Summer |
| Jorn | 8 | No | 0 | 2 | 59 | 19.00 | Spring |
| Kathrin | 7 | Yes | 1 | 0 | 12 | 25.34 | Autumn |
| Kjartan | 8 | Yes | 1 | 0 | 29 | 23.38 | Autumn |
| Kolbjorn | 3 | No | 0 | 1 | 15 | 18.50 | Summer |
| Kyle | 4 | Yes | 2 | 0 | 15 | 22.88 | Autumn |
| Kyle | 6 | Yes | 2 | 0 | 14 | 22.43 | Summer |
| Lars | 5 | Yes | 1 | 0 | 12 | 23.72 | Autumn |
| Lasse | 3 | No | 1 | 1 | 27 | 19.00 | Spring |
| Lasse | 7 | Yes | 1 | 1 | 25 | 22.88 | Spring |
| Laura | 2 | Yes | 1 | 0 | 14 | 20.88 | Spring |
| Laurits | 13 | Yes | 1 | 0 | 22 | 18.82 | Spring |
| Leaf | 6 | No | 1 | 1 | 6 | 24.70 | Autumn |
| Leaf | 3 | Yes | 1 | 1 | 15 | 20.88 | Autumn |
| Leigh | 12 | Yes | 1 | 0 | 18 | 22.63 | Spring |
| Leslie | 2 | No | 0 | 1 | 9 | 18.00 | Summer |
| Live | 5 | Yes | 1 | 0 | 8 | 23.35 | Autumn |
| Loran | 7 | No | 0 | 2 | 107 | 24.50 | Summer |
| Loran | 9 | No | 0 | 2 | 124 | 24.00 | Summer |
| Malena | 7 | No | 1 | 1 | 48 | 24.00 | Spring |
| Malena | 7 | Yes | 1 | 1 | 14 | 24.88 | Spring |
| Marta | 7 | No | 0 | 1 | 26 | 25.50 | Spring |
| Mason | 3 | Yes | 2 | 0 | 12 | 14.58 | Spring |
| Mason | 6 | Yes | 2 | 0 | 27 | 17.67 | Spring |
| Mattanja | 3 | Yes | 1 | 0 | 12 | 17.82 | Spring |
| Maximus | 2 | Yes | 1 | 0 | 13 | 16.92 | Spring |
| Mikkel | 7 | Yes | 1 | 0 | 17 | 25.22 | Summer |
| Moritz | 3 | No | 1 | 2 | 15 | 23.40 | Summer |
| Moritz | 3 | No | 1 | 2 | 133 | 21.40 | Summer |
| Moritz | 5 | Yes | 1 | 2 | 17 | 24.88 | Summer |
| Morten | 6 | Yes | 1 | 0 | 20 | 18.83 | Spring |
| Moses | 7 | Yes | 1 | 0 | 14 | 23.83 | Summer |
| Nanna | 5 | Yes | 1 | 0 | 20 | 22.88 | Spring |
| Odd Arne | 5 | Yes | 1 | 0 | 14 | 24.52 | Summer |
| Oddi | 5 | No | 0 | 1 | 44 | 18.00 | Spring |
| Orjan | 3 | No | 0 | 3 | 154 | 23.00 | Summer |
| Orjan | 5 | No | 0 | 3 | 86 | 24.00 | Summer |
| Orjan | 8 | No | 0 | 3 | 68 | 24.00 | Spring |
| Paddy | 12 | No | 3 | 1 | 57 | 25.30 | Summer |
| Paddy | 5 | Yes | 3 | 1 | 10 | 24.83 | Spring |
| Paddy | 6 | Yes | 3 | 1 | 13 | 25.83 | Autumn |
| Paddy | 7 | Yes | 3 | 1 | 23 | 25.33 | Spring |
| Randi | 5 | No | 0 | 1 | 29 | 20.50 | Summer |
| Rudolf | 6 | Yes | 1 | 0 | 14 | 20.88 | Spring |
| Sara | 4 | No | 0 | 1 | 36 | 22.50 | Spring |
| Solveig | 3 | Yes | 1 | 0 | 29 | 21.18 | Autumn |
| Sonja | 5 | No | 0 | 1 | 127 | 23.50 | Summer |
| Stina | 4 | No | 0 | 2 | 81 | 19.50 | Spring |
| Stina | 10 | No | 0 | 2 | 38 | 21.00 | Spring |
| Suzanne | 4 | No | 0 | 1 | 88 | 17.60 | Summer |
| Takehode | 4 | No | 0 | 2 | 91 | 20.00 | Spring |
| Takehode | 4 | No | 0 | 2 | 70 | 23.50 | Summer |
| Tanja | 13 | No | 2 | 1 | 117 | 19.50 | Summer |
| Tanja | 11 | Yes | 2 | 1 | 19 | 21.88 | Summer |
| Tanja | 15 | Yes | 2 | 1 | 22 | 18.42 | Summer |
| Terje | 7 | No | 0 | 1 | 119 | 20.00 | Summer |
| Thatcher | 8 | No | 0 | 1 | 93 | 23.00 | Summer |
| Thomas | 3 | No | 0 | 1 | 5 | 22.00 | Spring |
| Trude | 4 | No | 0 | 1 | 133 | 22.00 | Summer |
| Unni | 2 | No | 0 | 2 | 39 | 20.00 | Summer |
| Unni | 5 | No | 0 | 2 | 42 | 24.00 | Spring |
| Victoria | 4 | Yes | 1 | 0 | 15 | 22.08 | Spring |
| Waltraut | 6 | Yes | 2 | 1 | 13 | 24.58 | Autumn |
| Waltraut | 7 | Yes | 2 | 1 | 16 | 23.02 | Summer |
| Waltraut | 3 | No | 2 | 1 | 70 | 19.00 | Autumn |
| Yasmin | 4 | Yes | 1 | 0 | 14 | 22.68 | Spring |


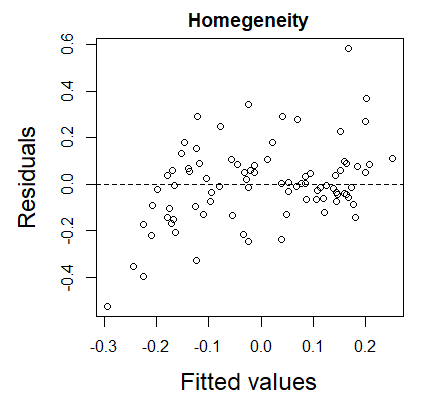


Fig. S1: Residuals versus fitted values from the most parsimonious model analyzing percentage daily body weight change between two subsequent captures of Eurasian beavers (*Castor fiber*) in southeastern Norway (2006-2020).


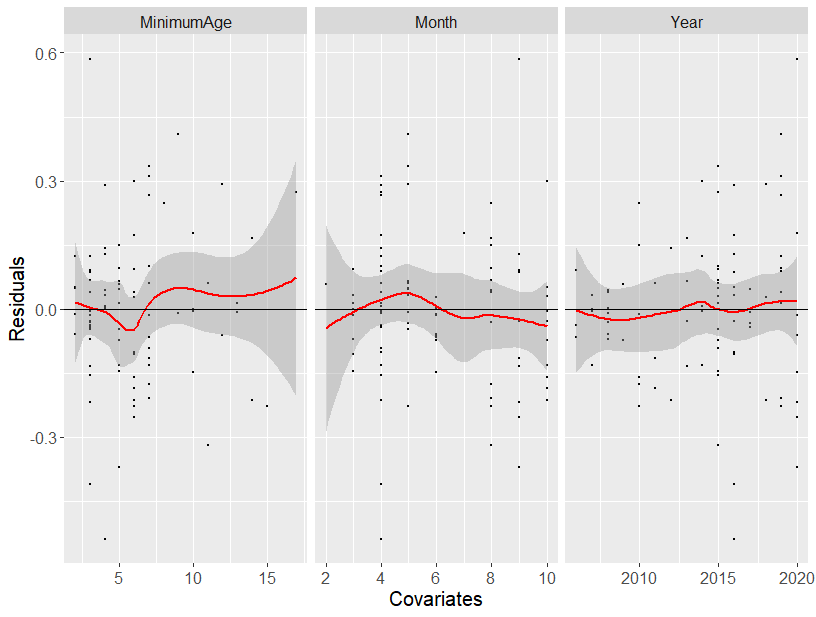


Fig. S2: Model residuals plotted against each numerical variable included and not included in the most parsimonious model analyzing percentage daily body weight change between two subsequent captures of Eurasian beavers (*Castor fiber*) in southeastern Norway (2006-2020). A smoother have been fitted to visualize any non-linear patterns in the residuals.


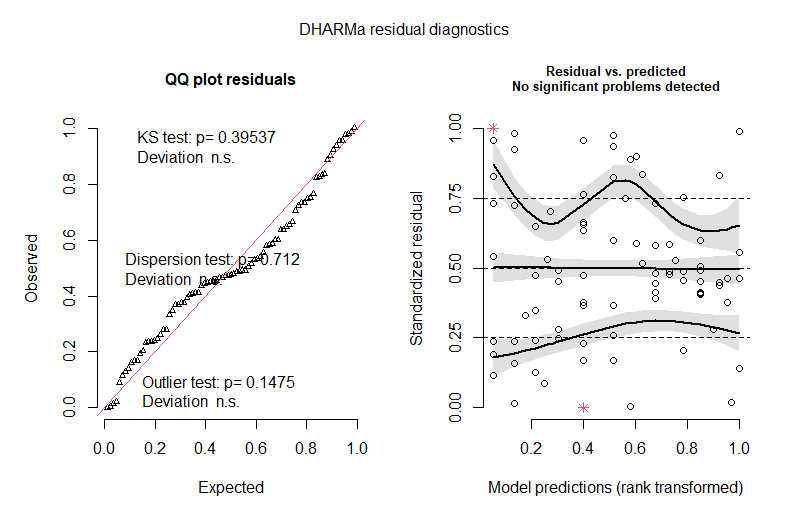


Fig. S3: Simulated residuals from model simulations using the DHARMa package from the most parsimonious model analyzing percentage daily body weight change between two subsequent captures of Eurasian beavers (*Castor fiber*) in southeastern Norway (2006-2020). The left panels show deviations from the expected distribution, while the right panel displays residuals against fitted values. The KS test results tests for whether the correct distribution is utilized.
